# Age-Related Decline in Expression of Molecular Chaperones Induces Endoplasmic Reticulum Stress and Chondrocyte Apoptosis in Articular Cartilage

**DOI:** 10.14336/AD.2019.1130

**Published:** 2020-10-01

**Authors:** Li Tan, Thomas C Register, Raghunatha R Yammani

**Affiliations:** ^1^Section of Molecular Medicine, Department of Internal Medicine, Wake Forest School of Medicine, Winston-Salem, NC 27157, USA; ^2^Departments of Pathology and Comparative Medicine, Wake Forest School of Medicine, Winston-Salem, NC 27157, USA

**Keywords:** aging, endoplasmic reticulum stress, apoptosis, cartilage, chaperones, osteoarthritis

## Abstract

Aging is a major risk factor for the development of osteoarthritis (OA). One hallmark of aging is loss of proteostasis resulting in increased cellular stress and cell death. However, its effect on the development of OA is not clear. Here, using knee articular cartilage tissue from young and old cynomolgus monkeys (*Macaca fascicularis*), we demonstrate that with aging there is loss of molecular chaperone expression resulting in endoplasmic reticulum (ER) stress and cell death. Chondrocytes from aged articular cartilage showed decreased expression of molecular chaperones, including protein disulfide isomerase, calnexin, and Ero1-like protein alpha, and increased immunohistochemical staining for ER stress markers (phosphorylated IRE1 alpha, spliced X-box binding protein-1, activating transcription factor 4 and C/EBP homologous protein), and apoptotic markers [cleaved caspase 3 and cleaved poly(ADP-ribose) polymerase], suggesting that decreased expression of molecular chaperone during aging induces ER stress and chondrocyte apoptosis in monkey articular cartilage. Apoptosis induced by aging-associated ER stress was further confirmed by TUNEL staining. Aged monkey cartilage also showed increased expression of nuclear protein 1 (Nupr1) and *tribbles* related protein-3 (TRB3), known regulators of apoptosis and cell survival pathways. Treatment of cultured monkey chondrocytes with a small molecule chemical chaperone, 4-phenylbutyric acid (PBA, a general ER stress inhibitor) or PERK Inhibitor I (an ER stress inhibitor specifically targeting the PERK branch of the unfolded protein response pathway), decreased the expression of ER stress and apoptotic markers and reduced the expression of Nupr1 and TRB3. Consistent with the above finding, knockdown of calnexin expression induces ER stress and apoptotic markers in normal human chondrocytes *in vitro*. Taken together, our study clearly demonstrates that aging promotes loss of proteostasis and induces ER stress and chondrocyte apoptosis in articular cartilage. Thus, restoring proteostasis using chemical/molecular chaperone or ER stress inhibitor could be a therapeutic option to treat aged-linked OA.

Osteoarthritis (OA) is the most common form of arthritis and the main cause of disability in older adults worldwide [1]. A progressive loss of articular cartilage is the hallmark of the disease process [2, 3]. Aging is the most significant risk factor for OA [4] and the mechanism involved are not clearly understood. The prevalence of radiographic knee OA in participants aged 45 and older increases with each decade of life from ~15% in the 45-54 age groups to ~50% in the 75+ age group [5]. Studies have reported that with aging several structural and molecular changes occur in articular cartilage including thinning of cartilage, decreasing glycosaminoglycan content [6], alterations of cartilage matrix components including aggrecan [7], and accumulation of advanced glycation end products [8]. Chondrocytes, the sole cellular component of articular cartilage, are responsible for the production of extracellular matrix proteins and maintenance of cartilage homeostasis. With aging, the capacity of chondrocytes to restore cartilage homeostasis declines due to aging-related cellular changes, including cellular senescence [9], oxidative stress [10], increased inflammation [11], autophagy defects [12] and mitochondrial dysfunction [13]. Taken together, these changes promote cartilage destruction and development of OA; however, the mechanisms involved are not clearly understood.

A key hallmark of aging is loss of proteostasis (protein homeostasis) due to decline in endoplasmic reticulum (ER) quality control machinery [14-16]. Maintaining cellular proteostasis requires stringent control of protein synthesis, folding/conformational maintenance, degradation, and export/elimination from the cell. Cells have assembled a complex network of molecular factors to coordinate these processes collectively called the proteostasis network (PN) [17]. Newly synthesized proteins are folded and modified in the ER before they are secreted or transported to their targeted destinations. Proteins are susceptible to many chemical and physical influences which may lead to unfolding and formation of abnormal non-physiologic aggregates. Molecular chaperones of various classes play a major role in keeping proteins in folded form and minimizing the formation of protein aggregate. Thus, molecular chaperone plays a major role in proteostasis [18].

Molecular chaperones are conserved proteins responsible for protein folding, degradation, and ER homeostasis [18]. Molecular chaperones are broadly categorized into multiple protein families: 1) heat shock proteins, 2) lectins (calreticulin and calnexin), 3) oxidoreductases or protein disulfide isomerases, and 4) co-chaperones that are members of ERp57 family that are associated with calnexin and calreticulin [19]. These chaperones play a very specific function in protein folding/proteostasis. For example, heat shock protein HSPA5 or binding immunoglobulin protein (BIP, also called GRP78) binds to nascent proteins and prevents their misfolding and aggregation. BIP also targets misfolded proteins for proteasomal degradation and acts as a repressor for unfolded protein response (UPR) signaling [20, 21]. The expression of BIP is directly associated with levels of misfolding or unfolded protein in the ER. Calnexin and calreticulin bind to specifically monoglycosylated protein moieties that are generated as a result from the trimming of two glucose residues by the sequential action of two glucosidases, glucosidase I and II, and provide aid in the proper folding of glycoproteins [22]. Protein disulfide isomerase (PDI) plays a major role in the formation of disulfide bonds between cysteine residues, a critical step for protein folding and assembly [23, 24]. PDI is the founder member of the PDI family that to date has 20 members. These proteins are identified by the presence of the thioredoxin-like domain at their active sites [25]. The members of PDI have oxidoreductase and isomerase properties supporting disulfide bond formation [25]. Additionally, they also bind to misfolded proteins and target them for degradation [26].

Loss of molecular chaperone activity and proteostasis cause accumulation of misfolded proteins in ER, resulting in ER stress. To counter the stress conditions, stress response pathways called UPR signaling is activated. UPR pathway is transduced by three branches of stress sensors: activating transcription factor 6 (ATF6), inositol-requiring enzyme 1 (IRE1), and protein kinase R-like ER kinase (PERK) [27, 28]. In healthy conditions, these sensors are bound to the ER chaperone BIP thus being dormant. Under stress conditions (e.g., accumulation of unfolded proteins), bound BIP releases itself from the sensors to chaperone unfolded proteins thus activating the cascade of signaling pathway to increase the expressions of molecular chaperones and shutting down the protein synthesis machinery of ER temporally. UPR is originally activated to restore ER/protein homeostasis. However, failure of this system results in severe and chronic stress condition leading to cell death [28, 29].

ER stress has been implicated in several aging-related diseases including type 2 diabetes, neurodegenerative diseases, certain types of cancer [29]. Elevated ER stress also contributes to aging-related inflammation in adipose tissue of mice [30]. Moreover, aging-induced ER stress regulates sleep homeostasis in fruit fly [31]. We recently demonstrate that obesity-related ER stress promotes apoptosis in cultured chondrocytes and meniscus cells *in vitro* [32, 33]. However, the role of ER stress in aging-related OA pathogenesis is poorly defined.

In this study, using non-human primate articular cartilage and isolated chondrocytes (monkey and human) we examined the effect of aging on the PN. Our data show that aged monkey articular cartilage/chondrocytes expressed low levels of molecular chaperones involved in ER protein folding apparatus and high levels of ER stress and apoptotic markers and that knockdown of calnexin (one of the tested molecular chaperones) expression induces ER stress and apoptosis in normal human chondrocytes, suggesting that ER stress induced by the loss of proteostasis in aged cartilage/chondrocyte might play a major role in aging-related OA.

## MATERIALS AND METHODS

### Monkey articular cartilage tissue

Knee articular cartilage tissues were obtained from five young (3 male and 2 female; 6-11 yrs) and six old (5 male and 1 female; 20-34 yrs) cynomolgus monkeys (*Macaca fascicularis*) at necropsy. These monkeys were part of other studies and have consumed western diets and standard chow during their lifetime at Wake Forest School of Medicine. Diet and water were provided *ad libitum*. Articular cartilage tissues were obtained from tibial plateau and graded for degenerative changes based on the five-point Collins scale [34]. Cartilage tissue from old monkeys did not show any signs of OA macroscopically. Unfortunately, we do not have imaging data to determine if older monkeys have any sign of early OA. Cartilage tissues were either fixed in 10% buffered formalin phosphate (Fisher Scientific) or digested to isolate chondrocytes as detailed below. Formalin fixed tissues were transferred to 70% ethanol after 24 hours. The fixed cartilage tissues were embedded in paraffin and sectioned at 5 µm. Cartilage sections obtained from the medial tibial plateau were selected for immunohistochemistry.

### Immunohistochemistry

Paraffin-embedded sections were deparaffinized in the xylene substitute (Clear Advantage, Polysciences), and rehydrated through a series of decreasing concentrations of ethanol, and then washed with Tris buffered saline (TBS). Antigen retrieval was achieved with proteinase K treatment for 5 min. Sections were washed with TBS, treated with 3% hydrogen peroxide for 15 min, washed with TBS, blocked with Vectastain? goat normal serum for 15 min at room temperature, and incubated with primary antibody [1:50 dilution for C/EBP homologous protein (CHOP), 1:100 dilutions for phosphorylated IRE1 alpha (P-IRE1α), activating transcription factor 4 (ATF4) and cleaved poly(ADP-ribose) polymerase (C-PARP), 1:200 dilutions for spliced X-box binding protein-1 (XBP1), nuclear protein 1 (Nupr1) and *tribbles* related protein-3 (TRB3), 1:500 dilution for BIP and cleaved caspase 3 (CC3)] in blocking serum overnight at 4°C. After washing with TBS, sections were incubated with biotinylated anti-rabbit secondary antibody for 30 min at room temperature, washed with TBS, and then incubated with Vectastain? Elite ABC reagent for 30 min at room temperature. Sections were again washed with TBS, incubated with ImmPACT™ NovaRED™ peroxidase substrate (Vector Laboratories) for 2-5 min, washed with TBS, dehydrated and mounted. The following antibodies were used: rabbit polyclonal anti-BIP (ab21685), rabbit polyclonal anti-ATF4 (ab105383), rabbit monoclonal anti-CHOP (ab179823), and rabbit monoclonal anti-C-PARP (ab32064), all from Abcam; rabbit polyclonal anti-P-IRE1α (PA1-16927 from Thermo Fisher Scientific), rabbit polyclonal anti-XBP1 (AP07389PU-N from OriGene Technologies); rabbit monoclonal anti-CC3 (9664 from Cell Signaling Technology), rabbit polyclonal anti-Nupr1 (bs-7106R from Bioss), and rabbit polyclonal anti-TRB3 (13300-1-AP from Proteintech). At least three immunohistochemical images/sections containing approximately 30-50 chondrocytes in the monkey knee cartilage were quantified using Adobe Photoshop CS6 (version 13.0) with correction for cell numbers.

### TUNEL staining

Chondrocytes apoptosis was further confirmed by TUNEL assay [35]. Paraffin-embedded sections were TUNEL stained according to manufacturer (Abcam) protocol and then visualized by Echo revolve fluorescence microscope and the data was quantified as descripted above.

### Chondrocyte isolation and culture conditions

Articular chondrocytes from monkey knee cartilage were isolated from tibial plateau region and cultured as described previously [32]. In brief, cells were isolated under aseptic conditions by sequential enzymatic digestion at 37°C using pronase at 2 mg/ml in serum-free Dulbecco’s modified Eagle’s medium/Ham’s F-12(1:1) (DMEMF) with antibiotics for 1 h, followed by overnight digestion with collagenase-P at 0.36 mg/ml in DMEMF containing 5% of fetal bovine serum (FBS). Viability of isolated cells was determined using trypan blue and cells were counted using a hemocytometer. High density monolayer cultures were established by plating cells in 12-well plates at 1 x10^6^ cells/well in DMEMF medium supplemented with 10% FBS at 37°C and 5% CO_2_. Cells were maintained for approximately 3 to 5 days with feedings every 2 days until they reached 100% confluence prior to experimental use.

### Chondrocyte transfection

Normal human cartilage tissue was obtained from the ankle joints of organ donors provided by the Gift of Hope Organ and Tissue Donor Network (Chicago, IL) through an agreement with Rush University Medical Center (Chicago, IL). Each cartilage specimen was graded for degenerative changes based on the five-point Collins scale [34]. Only cells from tissue graded as 0 or 1 (indicating no symptom of arthritis) were used in experiments. All the procedures and experimental protocols are approved by the Institutional Review Board (IRB) of the Wake Forest School of Medicine. The authors did not have access to any identifiable information and, as per the IRB that approved the study, no further consent was required. The ages of tissue donors ranged from 38 to 55 years. Chondrocytes were isolated and cultured as described above. Chondrocytes were transfected by nucleofection method using the Amaxa human chondrocyte nucleofector kit as described previously [32]. After transfection, cells were immediately transferred into warmed DMEMF/antibiotics containing 20% (v/v) FBS in 6-well plates previously coated with poly-lysine to help cell attachment, and incubated at 37°C and 5% CO_2_ overnight for recovery, then changed to DMEMF/antibiotics containing 10% (v/v) FBS overnight prior to cell treatment.

### Chondrocyte treatment and immunoblotting

Monkey chondrocyte (young and old) monolayers cultures were changed to serum-free media/antibiotics for 6 h followed by treatments with 4-phenylbutyric acid (PBA, a small molecule chemical chaperone and a general ER stress inhibitor) or PERK Inhibitor I (PERKi, an specific ER stress inhibitor targeting PERK branch of UPR pathway) overnight. For transfected human chondrocytes, cells were incubated in serum-free media/antibiotics for two days. After treatments, cell lysates were prepared as described previously [32]. Samples containing equal amounts of total protein were separated by sodium dodecyl sulfate-polyacrylamide gel electrophoresis and transferred to nitrocellulose for immunoblotting with anti-P-IRE1α, XBP1, ATF4, CHOP, Nupr1, TRB3, cytochrome c, CC3, PDI, calnexin, Ero1-like protein alpha (Ero1-Lα) and GAPDH antibodies, respectively. Immunoreactive bands were detected with the ECL western blotting detection reagents. Densitometry was performed on immunoblots using ImageJ software (National Institute of Health). All immunoblotting experiments were repeated with similar results using cells from three monkeys or human donors.

**Figure 1.**
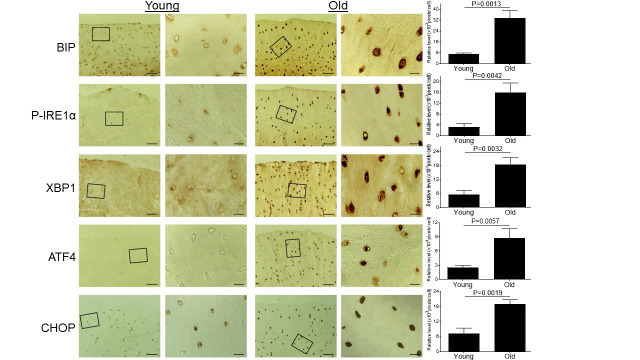
Aging induces ER stress in monkey knee cartilages. Articular cartilage sections of young (6-11 yrs, n=3) or old (20-34 yrs, n=3) monkeys were analyzed immunohistochemically for ER stress markers including BIP, P-IRE1α, XBP1, ATF4, and CHOP. Images on left panels are low magnification (Scale bars: 100 µm). The areas inside the small rectangles were magnified and displayed in right panels (Scale bars: 20 µm).

### PDI ELISA

PDI concentrations in young/old monkey chondrocyte lysates was further quantitated using a human PDI ELISA kit (MBS725967 from MyBioSource.com) according to manufacturer protocol.

### Statistical analysis

Data are expressed as mean ± standard deviation of at least three independent replicates. Statistical analysis of data was performed using GraphPad Prism 8 (version 8.2.0) as follows: a two-tailed unpaired *t*-test with exact P values for quantifications of immunohistochemical and TUNEL data as well as PDI ELISA data; two-tailed paired t-test with exact P values for densitometric analysis for immunoblotting data. The results were considered statistically significant at a value of P < 0.05.

## RESULTS

### Aging induces ER stress in monkey knee cartilages

Since ER stress has been implicated in multiple aging-related diseases [29], we wanted to test whether aging induces ER stress in articular cartilage. Cartilage tissue sections from knee joints of young and old monkeys were stained immunohistochemically for multiple ER stress protein markers. Our data showed significantly increased expression of ER stress markers, including BIP, P-IRE1α, XBP1, ATF4 and CHOP, in the articular cartilage of the old monkey compared to young monkeys (Fig. 1), demonstrating that aging induces ER stress in articular cartilage.

**Figure 2.**
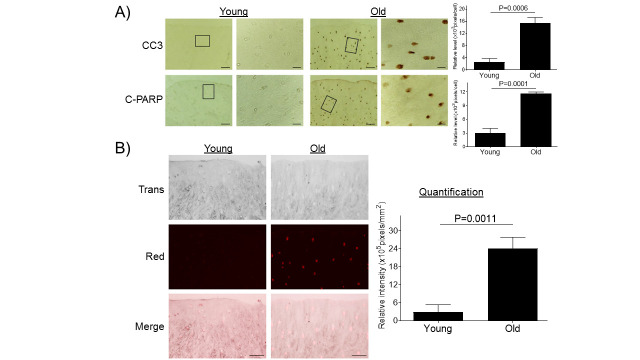
Aging promotes chondrocyte apoptosis in monkey knee cartilages. (A) Articular cartilage sections of young (6-11 yrs, n=3) or old (20-34 yrs, n=3) monkeys were analyzed immunohistochemically for apoptosis markers CC3 and C-PARP (A). Images on left panels are of low magnification (Scale bars: 100 µm). The areas inside the small rectangles were magnified and displayed in right panels (Scale bars: 20 µm). (B) Monkey knee cartilage sections were evaluated by TUNEL staining. Scale bar, 50 µm.

**Figure 3.**
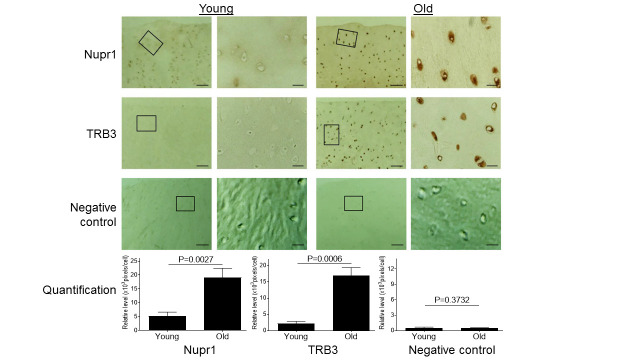
Aging induces increased expression of Nupr1 and TRB3 in monkey knee cartilages. Articular cartilage sections of young (6-11 yrs, n=3) or old (20-34 yrs, n=3) monkeys were analyzed immunohistochemically for Nupr1 and TRB3. Images on left panels are of low magnification (Scale bars: 100 µm). The areas inside the small rectangles were magnified and displayed in right panels (Scale bars: 20 µm). For the negative control, the cartilage sections were stained with only secondary antibody by replacing a primary antibody with the blocking serum.

### Aging promotes chondrocyte apoptosis

Severe and persistent ER stress induces caspase-mediated apoptosis [27, 36]. We investigated whether the aging-induced ER stress also triggers caspase-mediated apoptosis in articular cartilage. Immunohistochemical analyses of cartilage sections showed significantly increased expression of apoptosis markers CC3 and C-PARP [37] in cartilage tissue from old monkeys compared to young monkeys (Fig. 2A). Apoptosis was further confirmed by TUNEL staining as a functional assay to evaluate apoptosis-linked DNA fragmentation [35] (Fig. 2B). These results are consistent to previous findings showing a positive correlation between donor age and prevalence of chondrocyte apoptosis in articular cartilage [38].

### Aging induces increased expression of Nupr1 and TRB3

ER stress also induces the expression of Nupr1 and TRB3 that are known to play critical roles in cell survival and apoptosis [32, 39, 40]. Since both proteins were found to be increased in human OA cartilage [41, 42], we wanted to assess if aging-induced ER stress induced expression of Nupr1 and TRB3 in monkey articular cartilage. Compared to young monkey cartilage, old monkey cartilage showed significantly increased expression of both Nupr1 and TRB3 (Fig. 3), demonstrating that aging-induced ER stress stimulated expression of both Nupr1 and TRB3 protein in cartilage. As a negative control, immune-histochemical staining with only secondary antibody by replacing a primary antibody with the blocking serum essentially showed no or minimal staining for both young and old cells (Fig. 3).

**Figure 4.**
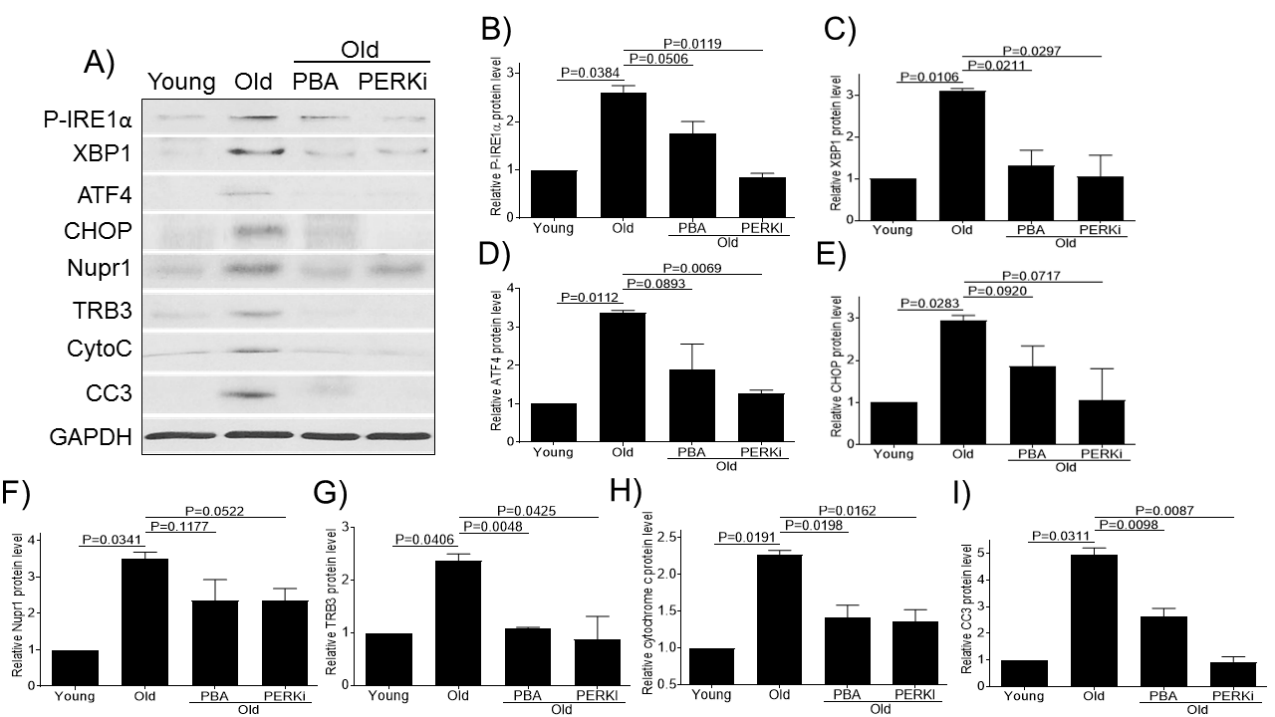
Small chemical chaperone PBA and PERK inhibitor alleviate ER stress and decrease chondrocytes apoptosis. Chondrocytes of young (6-11 yrs, n=3) or old (20-34 yrs, n=3) monkeys were treated with PBA or PERKi overnight and probed for P-IRE1α, XBP1, ATF4, CHOP, Nupr1, TRB3, cytochrome c, and CC3 antibodies, respectively (A). Blots were stripped and reprobed with GAPDH as a loading control. Densitometric analysis for protein levels of P-IRE1α (B), XBP1 (C), ATF4 (D), CHOP (E), Nupr1 (F), TRB3 (G), cytochrome c (H) and CC3 (I) were performed on blots obtained in independent experiments similar to the one shown in panel A. Data were shown as mean ± standard deviation of the mean. CytoC, cytochrome c.

### Small chemical chaperone PBA and PERK inhibitor alleviate ER stress and decrease chondrocytes apoptosis

Our above results indicate that aging induces ER stress and promotes chondrocyte apoptosis in monkey articular cartilage. We wanted to further examine whether a small chemical chaperone PBA (a general ER stress inhibitor that acts on all of three branches of UPR pathway) or PERKi (specifically targeted on the PERK branch of UPR pathway) could be used as an effective intervention to reduce ER stress and apoptosis *in vitro*. Consistent with the above immunohistochemistry results, the old monkey chondrocytes showed increased expression of ER stress (P-IRE1α, XBP1, ATF4, and CHOP) and apoptotic (cytochrome C and CC3) markers as well as increased expression of Nupr1 and TRB3 compared to young cultured chondrocytes (Fig. 4). Treatment of old chondrocytes with PBA or PERKi decreased the expression of Nupr1, TRB3, and ER stress and apoptotic markers (Fig. 4), indicating that both PBA and PERKi alleviate ER stress and reduces chondrocytes apoptosis.

### Aging reduces the expression of molecular chaperones required for proper protein folding

**Figure 5.**
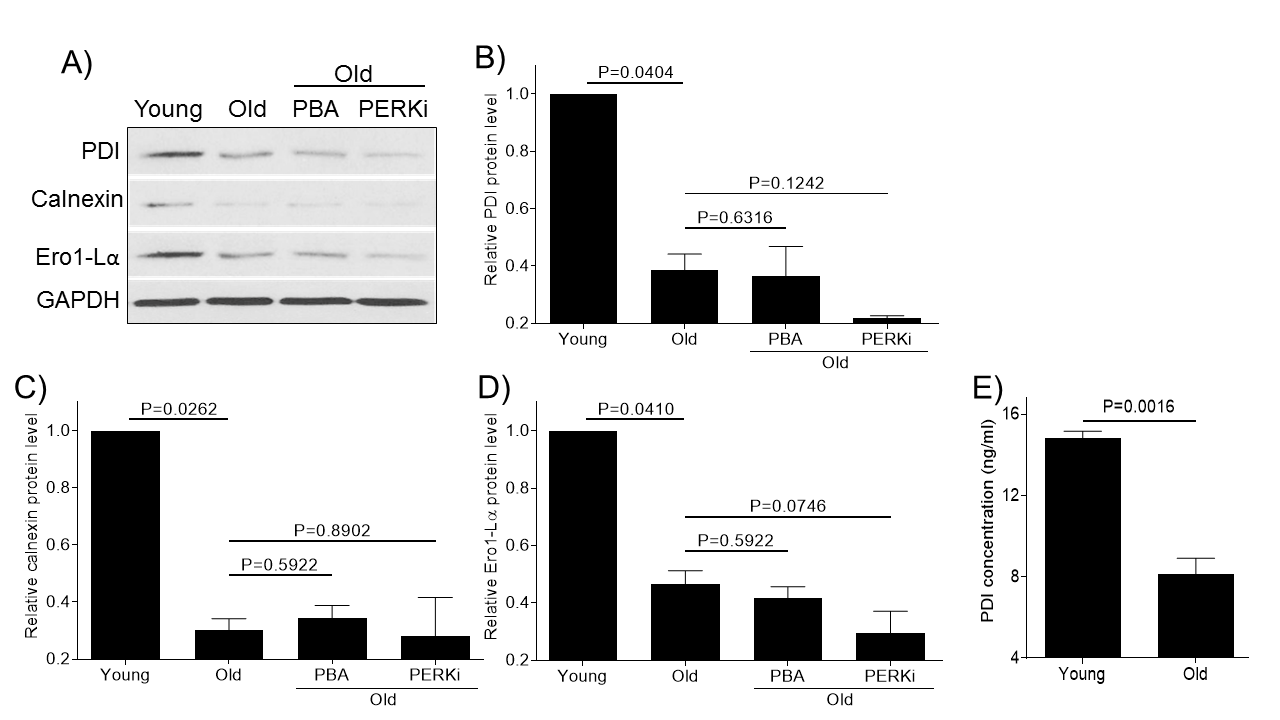
Aging reduces the expression of molecular chaperones required for proper protein folding. Chondrocytes of young (6-11 yrs, n=3) or old (20-34 yrs, n=3) monkeys were treated with PBA or PERKi overnight and probed for PDI, calnexin and Ero1-Lα antibodies, respectively (A). Blots were stripped and reprobed with GAPDH as a loading control. Densitometric analysis for protein levels of PDI (B), calnexin (C) and Ero1-Lα (D) were performed on blots obtained in independent experiments similar to the one shown in panel A. PDI concentrations in the young or old samples were further quantitated using a PDI ELISA kit (E). Data were shown as mean ± standard deviation of the mean.

Protein folding is a crucial step in maintaining protein stability, ER homeostasis and proteostasis and we wanted to test the effect of aging on the protein folding machinery. Articular chondrocytes from old monkey knee joint showed decreased levels of molecular chaperones (PDI, calnexin, and Ero1-Lα) by immunoblotting (Fig. 5A-D). The decrease of PDI levels in aged monkey chondrocyte was further confirmed by PDI ELISA (Fig. 5E). Treatment of old chondrocytes with PBA or PERKi did not have any significant effect on the molecular chaperone expressions (Fig. 5), consistent to their action locations either parallel or downstream to action sites of these chaperones.

### Knockdown of calnexin expression induces ER stress and chondrocyte apoptosis in normal human chondrocytes

**Figure 6.**
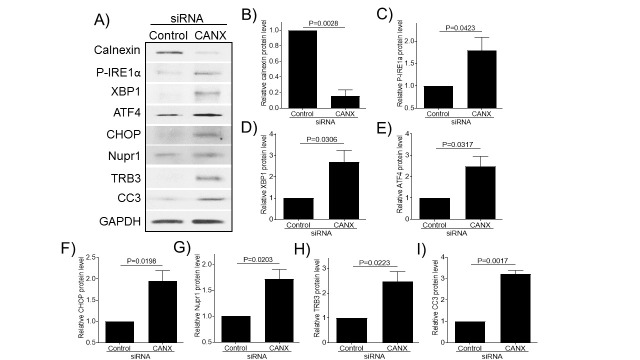
knockdown of calnexin expression induces ER stress and chondrocyte apoptosis in normal human chondrocytes. Human chondrocytes were transfected with control siRNA or siRNA specific for calnexin, and then were cultured in serum-free media for two days and cell lysates were probed for calnexin, P-IRE1α, XBP1, ATF4, CHOP, Nupr1, TRB3, and CC3 antibodies, respectively (A). Blots were stripped and reprobed with GAPDH as a loading control. Densitometry analysis for protein levels of calnexin (B) P-IRE1α (C), XBP1 (D), ATF4 (E), CHOP (F), Nupr1 (G), TRB3 (H), and CC3 (I) were performed on blots obtained in three independent experiments similar to the one shown in panel A. Data were shown as mean ± standard deviation of the mean. CANX, calnexin.

The above results strongly suggest that age-related decline in expression of molecular chaperones induces ER stress and chondrocyte apoptosis in articular cartilage. To further confirm, we knocked down calnexin expression by siRNA and examined its effect on the expression of ER stress and apoptosis protein markers in normal human chondrocytes (Fig. 6). Using calnexin-specific siRNA, we achieved an approximately 85% reduction in calnexin protein expression (Fig. 6A-B). Knocking down calnexin expression significantly increased the expression of ER stress [P-IRE1α (Fig. 6A, C), XBP1 (Fig. 6A, D), ATF4 (Fig. 6A, E), and CHOP (Fig. 6A, F)] and apoptotic [CC3 (Fig. 6A, I)] markers as well as increased the expression of Nupr1 (Fig. 6A, G) and TRB3 (Fig. 6A, H) compared to siRNA control, demonstrating that the loss of a molecular chaperone (e.g. calnexin) induces ER stress and apoptosis in both monkey and human chondrocytes.

## DISCUSSION

**Figure 7.**
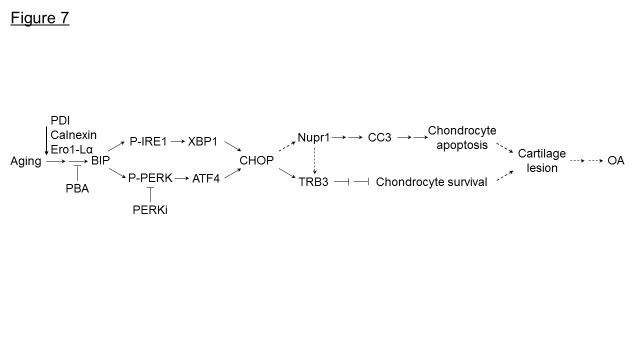
Model for aging inducing ER stress and chondrocyte apoptosis in monkey knee joints. Loss of molecular chaperones, (PDI, calnexin, Ero1-Lα) in aged monkey knee cartilage/chondrocytes induces ER stress. Under ER stress, BIP is dissociated from IRE1 to elicit activation of IRE1 via autophosphorylation (P-IRE1) which further induces the expression of spliced XBP1. Similarly, PERK is autophosphorylated (P-PERK) following the release of BIP thus inducing the ATF4 expression. Both XBP1 and ATF4 induce CHOP expression that further induces the expression of Nupr1 to trigger CC3-mediated chondrocyte apoptosis. Both CHOP and Nupr1 could induce TRB3 expression to inhibit chondrocyte survival. Treatment of aged monkey cartilage/chondrocytes with PBA or PERKi alleviates ER stress and reduces chondrocyte apoptosis. →, one-step stimulation; --->, putative one-step stimulation; → →, multistep stimulations; ---> --->, putative multistep stimulation; ?, one-step inhibition; ? ? , multistep inhibitions.

Age is a major risk factor for the development of OA. However, the mechanisms involved are not clear. Using a non-human primate model, here we demonstrate that with aging there was a decrease in protein homeostasis/proteostasis in articular cartilage resulting in chondrocyte apoptosis. Aged monkey articular cartilage had low levels of molecular chaperones including PDI, calnexin and Ero1-Lα, and increased ER stress levels compared to young cartilage. Furthermore, we also found that old articular cartilage expresses high levels of Nupr1 and TRB3, known regulators of cell death. Treatment of old chondrocytes with PBA, a small molecule chemical chaperone, or with PERKi, alleviated ER stress and decreased apoptosis. Furthermore, knockdown of calnexin expression induces ER stress and apoptosis in normal human chondrocytes. Taken together, our results suggest that restoring the cellular protein homeostasis alleviates ER stress and prevents chondrocyte apoptosis and may provide a novel therapy for aging-linked OA (Fig. 7).

Aging reduces the capacity of the cells to maintain protein homeostasis resulting in cellular stress (ER stress) and death [43]. Loss of proteostasis has been implicated in several age-related human pathologies, including Alzheimer’s Disease and Parkinson’s disease [44]; however, its role in OA pathogenesis is not clear. Protein folding is a critical and rate-limiting step in protein biogenesis mediated by chaperones and foldases [45], which have decreased efficacy with age [46]. Several mechanisms have been proposed for this decline in molecular chaperones activity, including oxidative [47] and mitochondrial dysfunction [48], changes that have been reported in aged chondrocytes/cartilage and in OA [49]. In our current study, we found that expression of PDI and Calnexin, two major proteins involved in protein folding in ER, was decreased in aged articular cartilage compared to young cartilage. In support of our data, studies have shown that expression and enzymatic activity of calnexin and PDI decrease with age [50, 51] and loss of their function results in the accumulation of misfolded proteins in the ER [52]. Furthermore, knocking down calnexin expression induced ER stress via IRE1 (IRE1 branch of UPR pathway) in transgenic mice [53], which is consistent to our finding (Fig 6). In our study, we found increased phosphorylation of IRE1alpha and increased expression of its downstream molecule XBP1 in old chondrocytes/cartilage compared to young tissue. Aged cartilage tissue also had high levels of ER stress markers, including ATF4 and CHOP, suggesting that loss of proteostasis induces ER stress in aged articular cartilage.

UPR signaling is activated to ease ER stress and restore ER homeostasis; however, under severe ER stress conditions, it promotes apoptosis [27]. UPR-induced apoptosis is mediated by IRE1/ASK/JNK pathway and CHOP. When exposed to unresolved/sever ER stress conditions, IRE1 recruits the adapter protein tumor necrosis factor receptor (TNFR)-associated factor 2 (TRAF2), apoptosis signal-regulating kinase (ASK1) and it’s downstream molecule JNK, a member of the mitogen-activated protein kinase family [36] and a known activator of apoptosis via caspase system [54]. We recently demonstrated that induction of ER stress in chondrocytes and meniscus cells activated JNK signaling pathways and promoted apoptosis. Alleviating ER stress or blocking IRE 1 pathways reduced ER stress-induced apoptosis [33, 55].

Another molecule that is induced during ER stress and plays a major role in ER-induced apoptosis is CHOP. CHOP is primarily induced by PERK arm of UPR; however, studies have shown that all three branches of UPR can induce CHOP [56]. CHOP induces apoptosis by suppressing the expression of Bcl-2, a pro-survival protein [57]. Induction of CHOP in chondrocytes promoted apoptosis and enhanced the catabolic effect of IL-1β [58]. Additionally, CHOP could also induce apoptosis via TRB3 [59]. TRB3 is a pseudokinase and a stress-inducible gene that is known to play a major role in cell survival [40, 59]. AKT signaling pathway is crucial for cell survival and studies have shown that TRB3 negatively regulates AKT signaling pathway [60]. Increased expression of TRB3 decreases phosphorylation of Akt at serine 473 and threonine 308 [60]. Activation of the Akt pathway blocks the apoptotic pathway by negatively regulating the activity of BAD, a member of the Bcl2 family that binds to Bcl2 or Bcl-XL and inhibits their anti-apoptotic activity [61], and the ASK1/JNK mediated pro-apoptotic pathway via phosphorylation of ASK1 at ser83 that inhibits ASK1 activity [62]. In this study, we found that aged cartilage had high levels of TRB3 expression compared to young cartilage. These studies show that TRB3 in conjunction with CHOP could play an important role in apoptosis.

Our studies also showed that aged cartilage/chondrocytes express high levels of Nupr1, a stress-inducible transcription factor we previously found to be upregulated in OA cartilage [42]. Nupr1 is known to play a major role in several cellular physiologic processes, including apoptosis [63]. We recently showed that CHOP is a key regulator of Nupr1 expression in chondrocytes, and that knocking down Nupr1 expression decreased TRB3 and CC3 expression and reduced apoptosis in human chondrocytes [32], suggesting that Nupr1 also plays a key role in CHOP-induced apoptosis in chondrocytes.

The limitation of this study was the lack of imaging data to determine if older monkeys have any signs of early OA. These monkeys were part of another study and unfortunately, their knee joints were not imaged.

In conclusion, our study shows that loss of chaperone activity with aging induced loss of proteostasis and ER stress, resulting in chondrocyte apoptosis in monkey knee cartilages. Treatment of old chondrocytes with a small chemical chaperone PBA or PERKi alleviated ER stress and reduced apoptosis. Therefore, our study establishes ER stress/UPR as potential therapeutic targets for aging-related OA.
